# Dr. Ozias Willard Peck

**Published:** 1916-10

**Authors:** 


**Dr. Ozias Willard Peck,** Yale 1857, died at his home in Oneonta, N. Y., Aug. 4, aged 81. He was a surgeon in the Civil War and was Health Officer of Oneonta for 26 years.

